# Coughs : Their Suppression and Cure

**Published:** 1901-04

**Authors:** 


### Abstracts.

### COUGHS: THEIR SUPPRESSION AND CURE.

Louis De Lorne, of New York, (*Interstate Medical Journal*,) says: The treatment of coughs in general has always been varied and unsatisfactory. With all of the numerous remedies of the materia medica, though each has had some particular value, there has always been the disadvantage of some unpleasant therapeutic effect following their use. Stomach disturbances, constipation, danger of habituation, and the like, are some of the complications experienced; especially are these conditions to be expected after the administration of codeine or morphine.

How especially important is it to have an efficient remedy for the cough of phthisis pulmonalis, which would not cause the disagreeable stagnation of secretory products of the lungs, that would not weaken the respiratory apparatus nor have any deleterious effect on the heart. Surely the profession has long felt the need of something to replace the various opiates for the suppression and cure of coughs, something that should be superior, more reliable and safer; a remedy that would not only be efficient, but safe for the treatment of the sympathetic cough of pregnancy; a remedy that can be used where heart complications occur; a remedy that will promptly check incessant, hacking cough, and paroxysmal coughs, which rob the patients of rest and sleep.

The complete clinical reports of Prof. Morris Manges, of the Mt. Sinai Hospital of New York, Dr. W. Freudenthal of New York, Dr. B. Turnauer (*Wiener Medizinische Presse*, No. 12, 1899,) Dr. A. E. Beketoff (*Klinische Therapeutische Wochenschrift*, No. 14, 1899,) following their investigation with heroin, I find to coincide with the results I have experienced with glyco-heroin (Smith), but I would here impress upon those interested that my entire investigations were made with glyco-heroin (Smith) only, excepting the few instances where I tried tablet triturates, and found them decidedly unsatisfactory.

Without doubt the merit of this preparation is to be attributed to its excellent composition, viz.: heroin, 1-16 grain; ammon. hypophosphite, 3 grains; hyoscyamus, 1 grain; white pine bark, 3 1-2 grains; balsam tolu, 1-4 grain, and glycerine sufficient to make 1 dram, wherein the therapeutic properties, if the drugs are properly compounded, and which is evidenced in this preparation, will be acknowledged desirable by every physician. Here I would point out the special advantage that this preparation possesses over heroin in any other form, viz.: the unnecessary administration of other remedies, while your patient is being treated with glyco-heroin (Smith), wherein you not only get the palliative effect of heroin, but have an admirable combination of remedies to bring about absolute cure in most cases. On account of the absence of syrup, instead of which glycerine, with its solvent and medicinal qualities, is used, there is not experienced the usual derangement of the stomach following the administration of preparations containing sugar or syrup. The addition of ammonia hypophosphite also appealed to me, for its superiority as an expectorant over other like remedies is an established fact and must add much to its therapeutic value. From the white pine bark contained therein the astringency of which is, no doubt, modified through the soothing effect of glycerine, I found a very beneficial effect on the mucous membranes, thereby proving this also a valuable and desirable addition.

The author then cited in detail a group of six cases in which he had administered glyco-heroin (Smith) for cough in various diseases and for various forms, giving intelligent and rational deductions therefrom, and closed his paper as follows:

And now, summing up all data of the clinical investigations carried on under my observation, I must say that we have here acquired a reliable, prompt and effective remedy with the following advantages: A reduction of temperature; prolonging respiration and at the same time increasing the volume of each respiration; almost devoid of hypnotic effect; absence of danger of acquiring the habit; it does not weaken the respiratory apparatus; does not cause unpleasant disturbances of the stomach or intestines; is without deleterious effect on the heart; the ratio of therapeutic to the toxic dose is many times smaller than that of morphine or codeine; it is of decided beneficial effect in all dyspnea; it does not constipate, or rarely, and then only slightly; it acts as a stimulant to the respiratory center, and stagnation of the secretions is excluded, and in comparative doses with codeine, the latter is shown to produce nausea and vertigo, while these symptoms are absent during the administration of glyco-heroin.

In conclusion, I would say that in all cases here cited, and many others as well, the instant relief obtained was a marvel both to myself and patients. I have never, with any remedy for similar indications, enjoyed the confidence I have in this preparation, and now have the pleasure in not only having offered relief and cure in the cases wherein this remedy was first suggested, but find glyco-heroin almost indispensable; and more especially so when I again consider codeine or morphine, with all their bad effects, as remedies compared with this preparation, which, on investigation, I found to be not only a true pharmaceutical product, but also an ethical product in every sense that the physician applies this term, in designating a medicinal preparation worthy of consideration by the medical profession.

